# Maternal influences on oral and faecal microbiota maturation in neonatal calves in beef and dairy production systems

**DOI:** 10.1186/s42523-020-00049-1

**Published:** 2020-09-07

**Authors:** Matthew Barden, Peter Richards-Rios, Erika Ganda, Luca Lenzi, Richard Eccles, Joseph Neary, Joanne Oultram, Georgios Oikonomou

**Affiliations:** 1 Department of Livestock and One Health, Institute of Infection, Veterinary & Ecological Sciences, University of Liverpool, Liverpool, UK; 2 Department of Veterinary Anatomy, Physiology and Pathology, Institute of Infection, Veterinary and Ecological Sciences, University of Liverpool, Liverpool, UK; 3 Department of Animal Science, The Pennsylvania State University, University Park, PA USA; 4 Department of Evolution, Ecology and Behaviour, Institute of Infection, Veterinary and Ecological Sciences, University of Liverpool, Liverpool, UK; 5 Department of Functional and Comparative Genomics, Institute of Systems, Molecular & Integrative Biology, University of Liverpool, Liverpool, UK

**Keywords:** Beef calves, Dairy calves, Microbiota, Neonates, Gastrointestinal tract

## Abstract

**Background:**

The dam is considered an important source of microbes for the calf; consequently, the development of calf microbiota may vary with farming system due to differences between the contact the calf has with the dam. The objective of this study was to characterise the early changes in the composition of oral and faecal microbiota in beef and dairy calves (*N* = 10) using high-throughput sequencing of the 16S rRNA gene. The microbiota of calves was compared to selected anatomical niches on their dams which were likely to contribute to the vertical transfer of microbes.

**Results:**

A total of 14,125 amplicon sequence variants (ASVs) were identified and taxonomically assigned. The oral microbiota of calves and their dams were composed of more similar microbes after the first 4 weeks of life than immediately after calving. The faecal microbiota of four-week old calves was composed of microbes which were more similar to those found in the oral microbiota of calves and adult cows than the faecal microbiota of adult cows. Specific ASVs were identified in the oral microbiota of four-week old calves that were also present in cow niches at calving, whereas very few ASVs were present in the calf faecal microbiota at four-weeks of age were present in any adult cow niche at calving. These results were observed in both beef and dairy calves.

**Conclusions:**

We did not observe any marked differences in the maturation of the oral and faecal microbiota between beef or dairy calves, despite dairy calves having very limited contact with their dam. This suggests the development of gastrointestinal microbiota in calves may not be affected by continued vertical transmission of microbes from the dam. Although the calf faecal microbiota changed over the first four-weeks of life, it was composed of microbes which were phylogenetically closer to those in the oral microbiota of calves and adult cows than the faeces of adult cows. There was little evidence of persistent microbial seeding of the calf faeces from anatomical niches on the cow at calving in either beef or dairy animals.

## Background

The gastrointestinal microbiota of the neonatal calf includes over 900 species of bacteria and varies both within and between populations [1–3]; the gastrointestinal microbiota has been associated with differences in growth rates, nutrient utilization and disease susceptibility [4–7]. Initial colonization of the gastrointestinal tract occurs during parturition due to exposure to microbes present in the dam’s faeces and vagina, followed by further opportunity for vertical transfer of maternal microbes when the dam licks the calf and when the calf feeds from the dam [1, 8, 9]. It is possible that the colonization of the bovine foetus occurs prior to parturition [8], but results are not definitive and the existence of an intra-uterine or placental microbiome is controversial in humans [10–14]. Following initial colonization, the gastrointestinal microbiota continues to develop, influenced primarily by environmental factors such as diet and exposure to antimicrobials [5, 15, 16], until it reaches a relatively steady state in adult animals [17, 18].

The vertical transmission of maternal microbiota to the neonate has been documented in many species in the animal kingdom [19]. The maternal influence on the early colonization of the infant gastrointestinal tract has been extensively described in humans [20–27]. Bacteria present in the mother’s faeces are considered the primary source of infant gastrointestinal microbiota [21, 25], but differences observed between infants born vaginally and by caesarean-section also implicate the vaginal microbiota as a potentially important source of microbes [28–30]. Vertical transfer between mother and infant has also been demonstrated via the teat-skin and breastmilk [31, 32]. In dairy cattle, studies have highlighted similarities between the neonatal gastrointestinal microbiota and the microbiota of the dam’s faeces, vagina, teat-skin, saliva and colostrum [1, 8, 9, 33]. In this context, similarities between dam and calf microbiota are interpreted as a likely indication of vertical microbial transfer. These studies report differences between which anatomical niches may play significant roles in the development of the neonatal microbiome.

Beef calves born on cow-calf suckler systems are left with the dam until weaning, whereas it is common practice for calves born on dairy farms to be removed from the dam following parturition and then housed separately and fed artificial milk replacer. The immediate removal of the calf from the dam in dairy production systems may therefore limit the vertical transfer of microbes. Differences have been reported in the diversity of the rumen microbiome between goat kids reared separately from the dam compared to kids kept with the dam [34]. This may be a consequence of feeding artificial milk replacer compared to the dam’s milk, and this has also been shown to influence the neonatal gastrointestinal microbiota in calves and humans [16, 35]. Therefore, management practices on beef and dairy farming systems could promote different microbiota development between beef and dairy calves.

The objectives of this study were to characterise the early maturation of oral and faecal microbiota in beef and dairy calves. Additionally, we compare the calf microbiota to select anatomical niches on their dams which may contribute to the vertical transfer of microbes.

## Results

### Study population

Samples were collected from five beef cows and five dairy cows over three time points, and from their respective calves on two occasions. The mean parity of beef cows at enrolment was 2.8 (range: 1–5) and 2.0 in dairy cows (range: 1–3). On the beef farm the average time from the first sampling point until calving was 30.4 days (range: 29–33 days) and on the dairy farm the average was 56.4 days (range: 53–60 days). The second sampling point was within twelve hours of calving in all cases (mean: 7.25 h). The average time between calving and the final sampling point was 26.6 days on the beef farm (range: 24–28 days) and 24.8 days on the dairy farm (range: 21–30 days).

### Sequencing results and quality control

The number of reads per sample are summarised in Supplementary Table 1. All DNA extraction negative controls were amplified and sequenced. No reads were observed in two of these negative control samples and the number of reads in other negative controls were generally very low (< 110 reads), except the calf oral negative control which had 508 reads. All negative control samples had considerably fewer reads than the majority of samples, therefore the degree of contamination during DNA extraction was considered very low and these sequences were not filtered out prior to analysis. Negative controls included during the PCR steps indicated that no contamination had occurred at this stage. The number of reads per sample was variable within and between sample types. Only one sample (milk) failed to produce any reads, while the remaining samples yielded a total of 7,177,398 reads following quality control. Excluding the negative controls and negative milk sample, the median number of reads per sample was 32,444 (IQR: 17,812). A total of 14,125 amplicon sequence variants (ASVs) were identified and taxonomically assigned. Across all samples, the average number of reads not classified was 8.3% at phylum level (SD: 2.34%), 9.9% at family level (SD: 23.8%); and 18.3% at genus level (SD: 22.3%). Taxonomic anlaysis was primarily conducted at family level because it would minimise the information lost due to unclassified samples at genera level, although descriptions at this level may slightly inflate similarities between samples. The number of unique ASVs per sample ranged from 9 to 2691 (median: 432, IQR: 446).

### Taxonomy

**Fig. 1:**
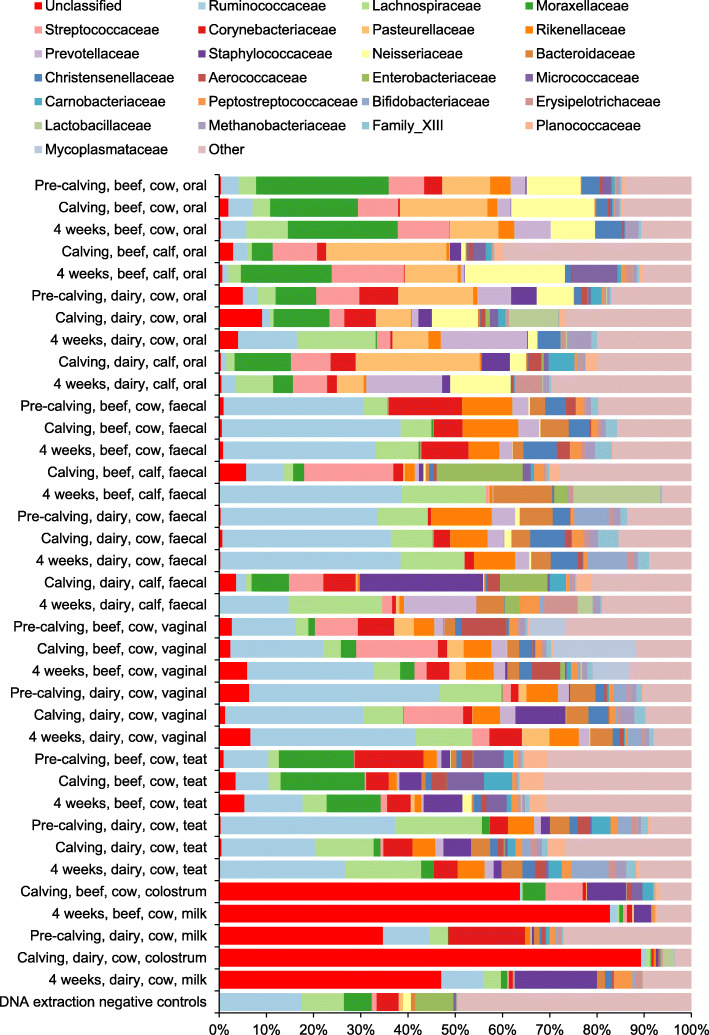
The relative abundance of taxonomic family in each group of samples. The mean relative abundance of the most frequently classified families across all samples. Less frequently identified families are grouped as “Other”. Unidentified families are grouped as “Unclassified”. The number of individual animals in each group: each sample *N* = 5 unless no reads were produced, therefore “DNA extraction negative controls” *N* = 6 and “4 weeks, dairy, cow, milk” *N* = 4

The relative abundance of the 25 most common families across all samples is displayed in Fig. 1; summary statistics of the relative abundance of each family in all samples are provided in Additional Data 1. Changes in average relative abundances are just presented descriptively, changes over time and comparisons between groups should be interpreted with caution due to the small number of individuals in each group. We did not conduct statistical tests to assess changes in relative abundance due to the low statistical power, inter-individual variability, and large number of relevant comparisons [36]. The most abundant phyla in the oral and faecal microbiota of both cows and calves were Proteobacteria, Firmicutes, Actinobacteria and Bacteroidetes; the changes in the relative abundance of these phyla between calving and four-weeks are displayed in Supplementary Fig. 1.

Proteobacteria was the most abundant phylum in the oral microbiota of calves. Within this phylum, *Pasteurellaceae* had the highest mean relative abundance in both beef and dairy calves at calving. By four weeks there was a marked decrease in its average relative abundance in dairy calves (26.3 to 5.7%) which was less substantial in beef calves (25.5 to 11.3%). The overall, average relative abundance of *Pasteurellaceae* in the oral microbiota of adult cows was 11.7%. The overall relative abundance of *Moraxellaceae* in the oral microbiota of adult cows was 23.3% in beef cows and 6.9% in dairy cows. There was a marked increase between calving and four-weeks in beef calves (4.4 to 19.2%) whereas the average relative abundance in dairy calves decreased from 11.9 to 4.2%. Another family in the Proteobacteria phylum, *Neisseriaceae* (predominantly *Alysiella* genus), generally had a higher overall relative abundance in adult beef cows (12.8%) than adult dairy cows (6.5%) and also showed a much greater increase over the first four weeks of life in beef calves, 1.0 to 21.1%, than observed in dairy calves, 3.5 to 12.8%.

In calf faeces, the relative abundance of Proteobacteria decreased between calving and four-weeks of age. This change was driven in part by changes in the relative abundance of *Enterobacteriaceae*, particularly *Escherichia-Shigella.* This genus had a mean relative abundance of 18.1% in beef calves and 9.5% in dairy calves at calving, but dropped by 4 weeks to 2.8 and 3.2% respectively; it was considerably lower in all adult faecal samples (< 0.05%).

Firmicutes was the predominant phylum in the faecal microbiota of calves and adult cows. In calves, the most abundant family within the Firmicutes phylum at calving was *Streptococcaceae* (relative abundance: 18.9%) in beef calves and *Staphylococcaceae* (relative abundance: 26.1%) in dairy calves. By four-weeks of age both of these families had markedly decreased: *Streptococcaceae* was 0.6% in beef calves and 2.2% in dairy calves; *Staphylococcaceae* was absent in both beef and dairy calf faeces. Overall, the most abundant family in adult faecal samples was *Ruminococcaceae* (34.5%); it was present in a lower relative abundance at calving in both beef and dairy calves (8.0 and 2.2% respectively). After four weeks, *Ruminococcaceae* had increased in beef calves to 38.7%, a similar relative abundance to adult cows, but only to 14.7% in dairy calves. Another family in the Firmicutes phylum, *Lachnospiraceae*, was present at an average relative abundance of 8.9% in the faeces of adult cows and increased comparably in both beef and dairy calves from an initially low relative abundance (< 2%) to 17.8 and 19.8% in beef and dairy calves respectively.

The colostrum samples and particularly milk samples tended to have a low number of reads compared to other samples (Supplementary Table 1). Furthermore, these samples had a higher proportion of unclassified reads than other sample types (Fig. 1), therefore the relative abundance of families in these samples should be interpreted with particular caution. The families that were present at a relative abundance > 2% in more than one milk or colostrum sample were *Staphylococceae* (mean: 5.4%), *Ruminococcaceae* (mean: 4.0%) and *Lachnospiraceae* (mean: 1.7%).

*Moraxellaceae* was found in a high relative abundance on the teat-skin of beef cows (mean: 15.0%) but not dairy cows (mean: 1.9%). *Ruminococcaceae* were present in high relative abundances on the teat-skin of all adult cows although higher in dairy cows than beef cows: 27.8 and 9.6% respectively. *Ruminococceae* was the family with the highest average relative abundance in the vaginal samples of all cows, but it was higher in dairy cows (mean: 34.9%) than beef cows (mean: 20.0%). *Streptococcaceae* was present at the highest average relative abundance in the vaginal microbiota of freshly calved beef and dairy cows, 17.3 and 12.6% respectively, compared to the pre- and postpartum time points where the average relative abundance was 4.3%.

The archaea family with the highest relative abundance was *Methanobacteriaceae* which was found at the highest relative abundances in the oral, vaginal and faecal microbiota of adult cows: 1.6%; 1.3, and 1.4% respectively. It increased in the oral microbiota of calves over the first four weeks of life in both beef (0.04 to 0.8%) and dairy (0.5 to 1.2%) calves, and it increased in the faeces of dairy calves (0.2 to 1.6%) but not beef calves (0.1% at both time points).

### Diversity analyses

**Fig. 2:**
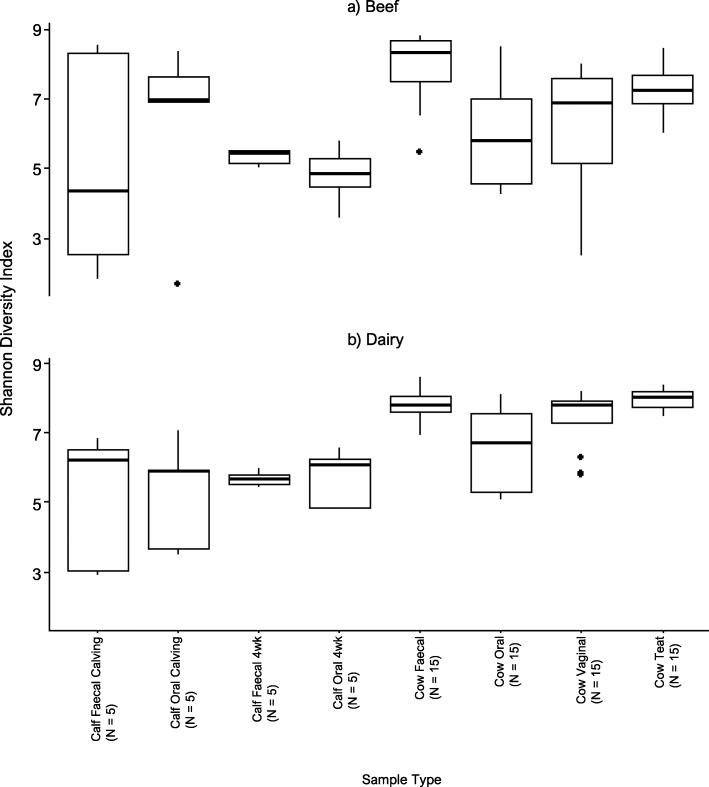
Shannon diversity index for each sample in beef (**a**) and dairy (**b**) animals. Beef and dairy animals are plotted separately, faecal and oral samples from calves are compared between “Calving” (within 12 h of parturition) and “4 weeks” (four-weeks of age). Samples from adult cows are displayed as an average across all three timepoints (six to eight weeks prepartum, within 12 h of parturition, and four-weeks postpartum). The number of individual animals in each group: calf samples *N* = 5 and adult cow samples *N* = 15

Rarefection curves demonstrated a pleateau in Shannon diversity index at approximately 12,000 sequences per sample, therefore we excluded samples below this threshold from subsequent alpha- and beta-diversity anlayses. Alpha-diversity was assessed by calculating the Shannon diversity index for each sample (Supplementary Table 1). The rarefection threshold excluded all but two milk and two colostrum samples resulting in small numbers in these groups, therefore these sample types were excluded from further analysis. Due to the small number of animals in each group, the average and distribution of alpha-diversity in each sample type are displayed (Fig. 2), but pairwise statistical comparisons were considered to be of limited value.

**Fig. 3:**
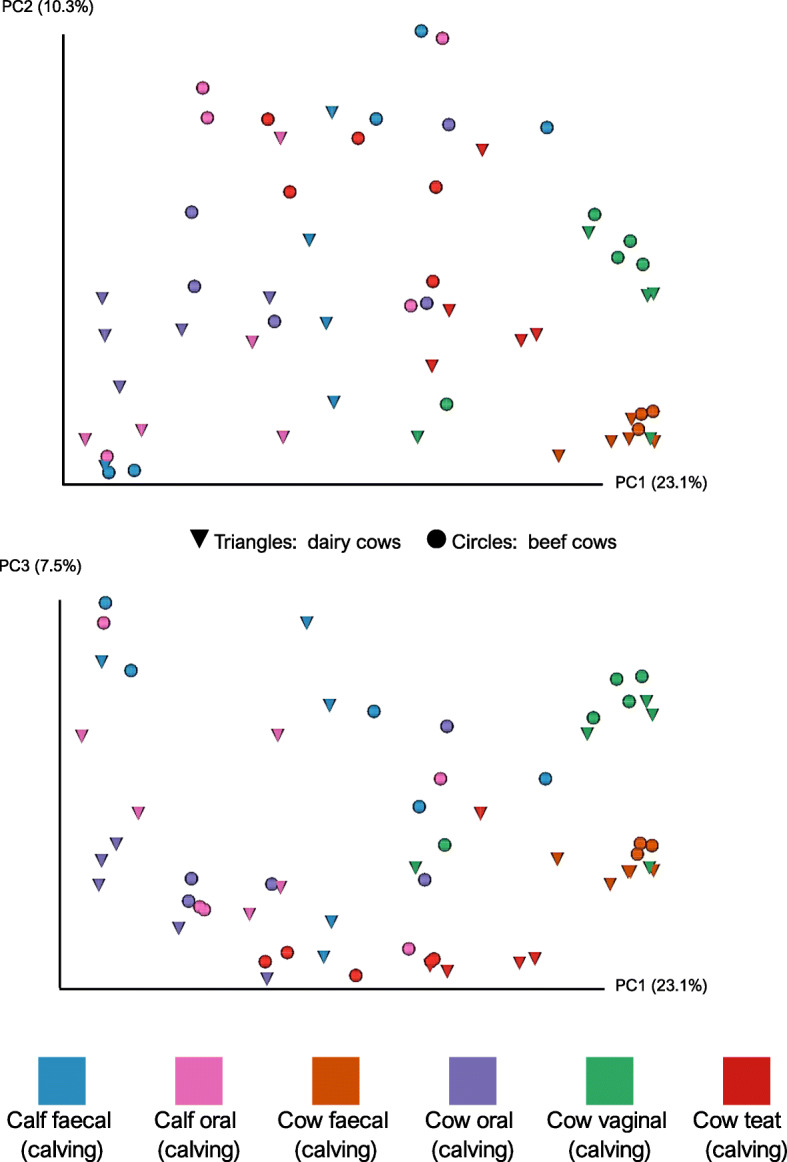
Principal Coordinate Analysis (PCoA) of unweighted UniFrac distances at Calving. Beef animals are represented by circles and dairy animals by triangles. The “Calving” timepoint is up to 12 h after parturition. The two plots display the same data but with different y-axes to display the three-dimensional relationship between datapoints. The number of individual animals in each group: N = 5

**Fig. 4:**
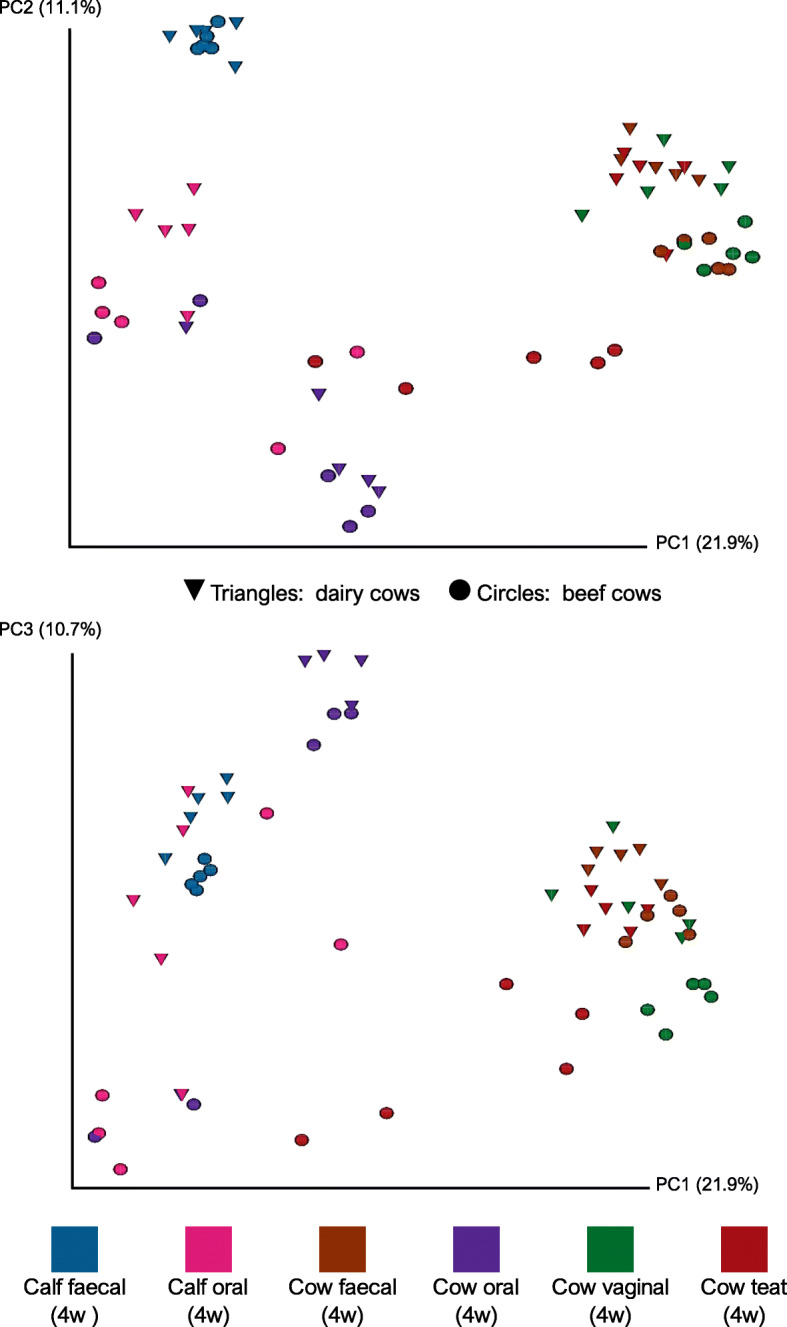
Principal Coordinate Analysis (PCoA) of unweighted UniFrac distances at the four-weeks. Beef animals are represented by circles and dairy animals by triangles. The four-week timepoint is 4 weeks after parturition. The two plots display the same data but with different y-axes to display the three-dimensional relationship between datapoints. The number of individual animals in each group: N = 5

The beta-diversity between samples was assesed by calculating weighted and unweighted UniFrac distances, and Bray-Curtis dissimilarities. Preliminary Principal Coordinates Analysis (PCoA) included the milk and colostrum samples which exceeded the rarefaction threshold and indicated that there appeared to be a difference between the microbiota of the teat skin and milk/colostrum (Supplementary Fig. 2). Milk and colostrum samples were then excluded, and the PCoA repeated which allowed better appreciation of sample clustering. Unweighted UniFrac distances were chosen as the primary metric of beta-diversity because they directly addressed the study objective to assess potential microbial sharing between cows and calves. Futhermore, this is an appropriate metric to compare samples from different microbial environments. PCoA using unweighted UniFrac distances displayed clear clustering of samples, which illustrated the qualitative phylogenetic similarities between different sample types (Figs. 3 and 4). PCoA using weighted UniFrac distances were skewed by a small number of samples. These outlying samples were not filtered out for this analysis because they included faecal samples from newborn calves and therefore would have undermined the aims of this study.

In newborn calves the faecal and oral microbiota were loosely clustered but became more closely related at four-weeks of age. At four-weeks of age the faecal microbiota of beef calves, showed very little inter-sample variability and distinct clusters of beef and dairy calves were apparent. The microbes in the faecal microbiota of calves appeared to be phylogenetically different from those constituting the faecal microbiota of adult cows. Vaginal microbiota of adult cows appeared to cluster, but more diffusely than the faecal microbiota which clustered tightly. The teat samples from beef and dairy cows formed distinct clusters, and the teat skin microbiota appeared to be similar to faecal microbiota from dairy cows, but this was less apparent in beef cows.

Permutational analysis of variance (PERMANOVA) of weighted and unweighted UniFrac distance matrices between animal type and age, sampling timepoint and sample type indicated significant differences which are demonstrated by the clustering in PCoA plots (weighted UniFrac distances: pseudo-F = 6.452, *P* value = 0.001; unweighted UniFrac distances: pseudo-F = 46.025, P value = 0.001). Pairwise statistical comparisons in beta-diversity metrics were not conducted for reasons outlined previously, namely: small sample size, inter-individual variability, and large number of relevant comparisons.

### Set analysis

**Fig. 5:**
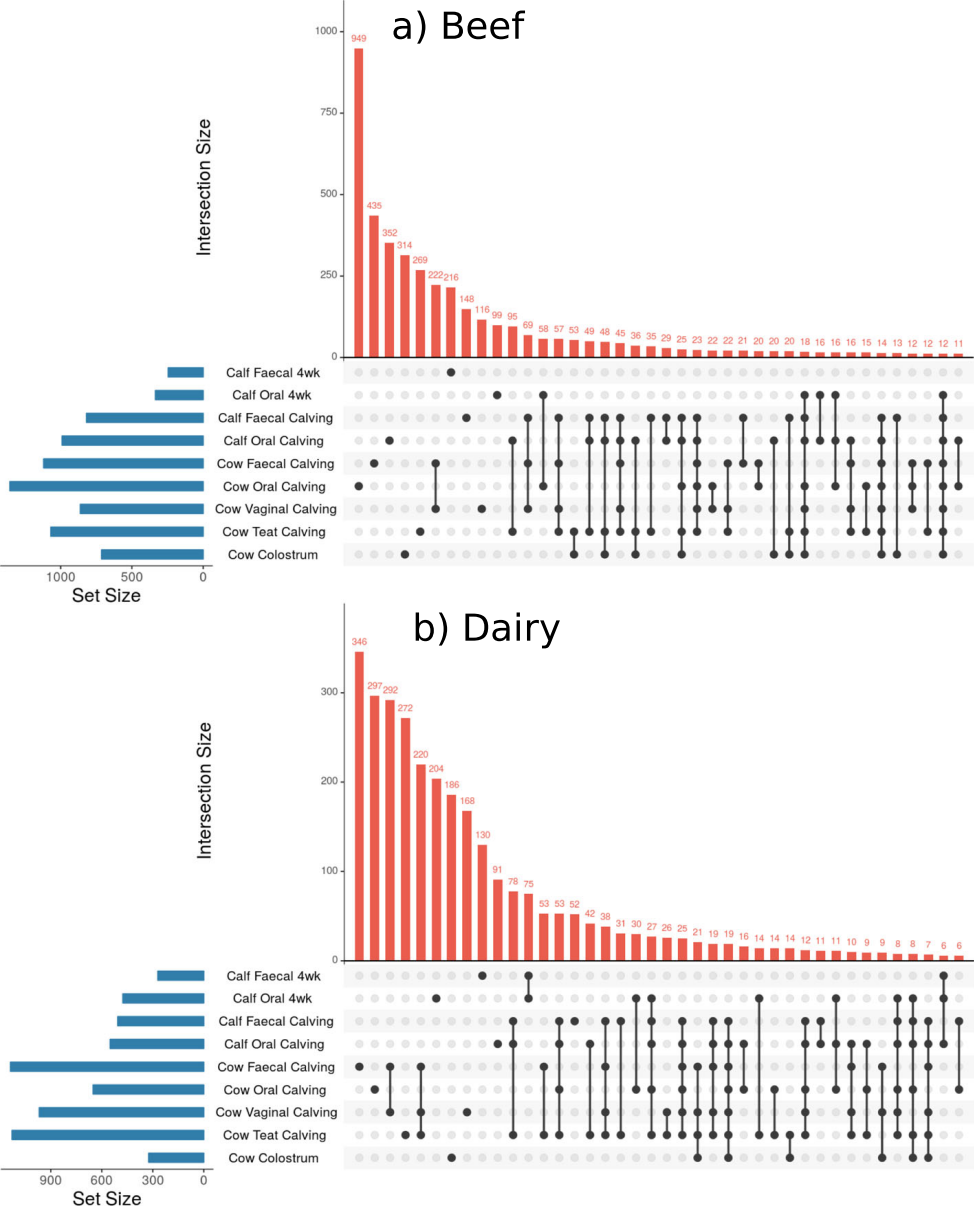
UpSet plots of common assigned sequence variants (ASVs) between samples. Beef (**a**) and dairy (**b**) animals are displayed separately. All cow samples collected at “Calving” (within 12 h of parturition) and the oral and faecal samples from calves collected at “Calving” and “4wk” (four-weeks of age) are displayed. Only ASVs with an overall abundance across all samples of greater than 0.01% are included, the 30 intersections which involve the greatest number of ASVs are displayed. The number of individual animals in each group (set): *N* = 5. Taxonomy of each intersection is detailed in Supplementary Table 2 and 3

**Fig. 6:**
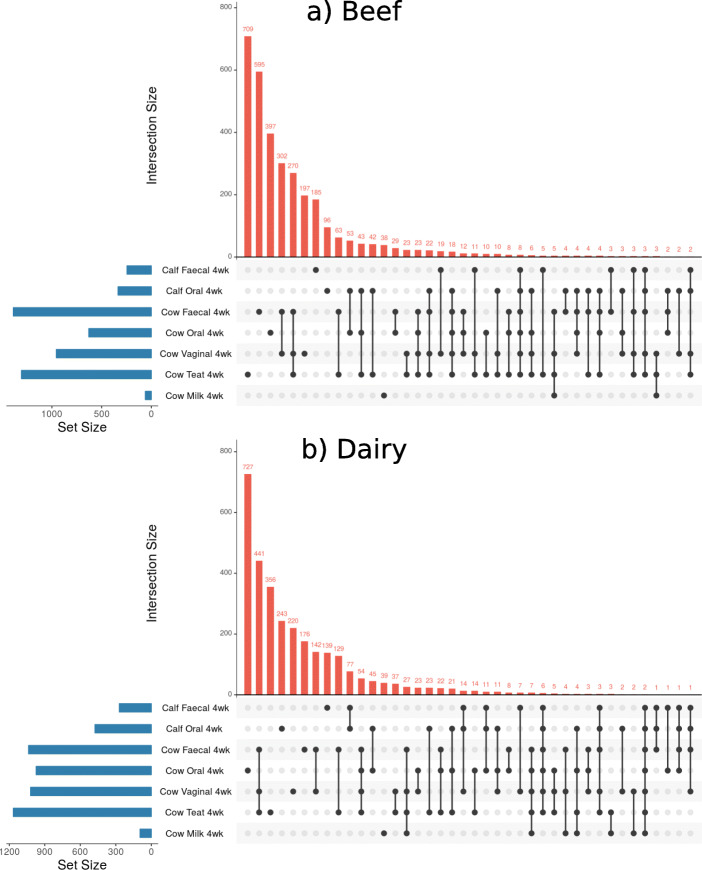
UpSet plots of common assigned sequence variants (ASVs) between samples at 4 weeks postpartum. Beef (**a**) and dairy (**b**) animals are displayed separately. All samples collected at “4wk” (four-weeks postpartum) are displayed. Only ASVs with an overall abundance across all samples of greater than 0.01% are included, the 30 intersections which involve the greatest number of ASVs are displayed. The number of individual animals in each group (set): N = 5. Taxonomy of each intersection is detailed in Supplementary Table 4 and 5

Set analysis was performed to determine common ASVs in different sample types limited to ASVs with a relative abundance above 0.01% across samples of that type. The shared ASVs between sampling sites are displayed in Figs. 5 and 6 and Supplementary Fig. 3. The families represented by the ASVs in these intersections are provided in Supplementary Tables 2, 3, 4, 5, 6, 7.

There were ASVs identified in the oral microbiota of beef and dairy calves at four-weeks old which were also present in anatomical niches of the cow at calving, with the exception of dairy cow faeces. The oral microbiota of calves at four-weeks of age had a large number of ASVs which were also present in the cow oral microbiota at calving, but no other niches. In beef animals, the most prevalent families represented by these common ASVs were *Streptococcaceae* and *Pasteurellaceae;* these families had a mean relative abundance in the oral microbiota of four-week old calves of 15.3 and 11.2% respectively. In dairy animals the most prevalent family represented by these common ASVs was *Neisseriaceae*, followed by *Streptococcaceae* and *Pasteurellaceae;* these families had a mean relative abundance in the oral microbiota of four-week old dairy calves of 12.8, 7.3, and 5.7% respectively. A smaller number of ASVs were exclusively present in the oral microbiota of calves at four-weeks of age and the oral microbiota of both cows and calves at calving. In beef animals, the most represented family in this intersection was *Moraxellaceae*, which had a relative abundance of 19.2% in the oral microbiota of four-week old calves; in dairy animals the most represented family was *Streptococcaceae.*

At calving there were a large number of ASVs that were common to the microbiota of calf faeces and the adult cow vagina, faeces, and teat-skin. In both beef and dairy animals these ASVs were dominated by *Ruminococcaceae* and *Rikenellaceae* families. These ASVs were not present in the faecal microbiota of four-week old calves, which had very few, if any, ASVs which were also present in adult cow niches at calving.

## Discussion

The objectives of this study were to characterise the early maturation of oral and faecal microbiota in beef and dairy calves, and to highlight if microbes may have been shared between anatomical niches on the dam and their calves. By four-weeks of age, the oral microbiota of calves was composed of microbes which were more similar to those found in the oral microbiota of adult cows, whereas the faecal microbiota was composed of microbes which bore little resemblance to those in the faeces of adult cows. This was similar in both beef and dairy calves despite dairy calves having no contact with their dam after the first few hours of life. This suggests the development of the gastrointestinal microbiota in calves may not be dependent on continued maternal contact.

### Oral microbiota changes in calves and cows

The oral microbiota of newborn calves changed in the first four weeks of life to contain microbes which were similar to those found in the oral microbiota of adult cows. This was also described by Alipour et al. (2018) in calves which were separated from the dam 24 h after parturition [8]. In our study, this trend occurred in both beef and dairy calves and therefore did not appear to be affected by whether or not the calf was separated from the dam. PCoA indicated there were separate beef and dairy clusters in oral microbiota at four-weeks of age (Fig. 4), but both appeared to contain microbes which were similar to those in the adult cow oral microbiota. Additionally, set analysis indicated that in beef and dairy calves, the oral microbiota at four-weeks of age had a large number of ASVs exclusively in common with the cow oral microbiota at calving (Fig. 5), which included families which had the greatest relative abundance in the calf oral microbiota such as *Neisseriaceae, Streptococcaceae and Pasteurellaceae.* These differences also support the assertion that the oral microbiota of calves progresses towards the adult microbiota over the first four weeks of life, in a similar manner in both beef and dairy calves. The number of ASVs that were present exclusively in both the four-week old calf oral microbiota and the oral microbiota of the cow at calving was greater in beef animals than dairy animals. This may suggest that more microbial transfer occurred between the cow and calf oral microbiota in beef animals than dairy animals immediately postpartum, possibly reflecting the reported differences in mothering behaviours between beef and dairy breeds [37].

The similarity between cow and calf oral microbiota appeared to be loosely dependent on the sampling time point, although there was a moderate degree of inter-animal variation in these samples. The oral microbiota of the newborn calf contained microbes which were most similar to those in oral microbiota of the cow at calving, whereas at four-weeks of age the calf oral microbiota constituted microbes were more similar to those in the cow oral microbiota at four-weeks postpartum, and indeed 4–8 weeks prepartum. There were a large number of ASVs in the oral microbiota of adult cows at calving which were not present in the oral microbiota in pre- or postpartum samples (Supplementary Fig. 3). This trend could suggest that the newborn calf alters the oral microbiota of adult cows in the periparturient period, although the oral or faecal microbiota of the calf do not appear to influence this, or the oral microbiota of the cow changes for another reason.

### Oral microbiota of calves and teat-skin microbiota of cows

The changes in the relative abundances of *Moraxellaceae* in the calf oral microbiota over the first four weeks of life were different in beef and dairy calves (4.4 to 19.2% in beef calves; 11.9 to 4.2% in dairy calves). One explanation for the relative abundance of *Moraxellaceae* increasing in the oral microbiota of beef calves, but decreasing significantly in dairy calves, is the continued sharing of microbes between the cow teat skin and calf oral cavity in beef cows through suckling. This is supported by the set analysis which indicated that *Moraxellaceae* was the most prevalent family represented by the ASVs common to both the cow teat skin and calf oral microbiota*.* The teat-skin of beef cows had a high relative abundance of *Moraxellaceae* which was also high prepartum and therefore more likely to a be source of *Moraxellaceae* in the oral microbiota of the calves, rather than a consequence. The relative abundance of *Moraxellaceae* on the teat-skin of dairy cows was low, consistent with previous reports [38, 39].

### Faecal microbiota changes in calves

The faecal microbiota of calves changed significantly in the first 4 weeks of life, consistent with previous studies [5, 6, 8, 9]. However, we observed limited phylogenetic similarity between microbes in the faecal microbiota of calves and adult cows (Figs. 3 and 4). Furthermore, the general changes in the faecal microbiota of calves were similar in both beef and dairy animals, suggesting they may not be influenced by continued cow-calf contact.

There was a decrease in beta-diversity of faecal microbiota in beef and dairy calves by four-weeks of age, as has been previously reported [5, 17, 33]. In general, microbes in faecal microbiota of all calves at four-weeks of age were most similar to those in the oral microbiota of four-week old calves and adult cows. This is consistent with a previous study [8], in which authors suggest the oral microbiota of the cow seeds the faecal microbiota in calves. However, the set analysis indicated that few, if any, ASVs present in the cow oral microbiota at calving persisted in the calf faeces 4 weeks later. Nevertheless, the phylogenetic similarity between components of the calf faecal and adult oral microbiota appeared to be comparable in both beef and dairy calves. This suggests that if seeding from cow to calf does occur, it occurs in the first few hours after parturition before dairy calves are separated.

More dairy calves had a faecal microbiota that consisted of microbes similar to those in their oral microbiota at four-weeks of age than beef calves, the same trend was also evident in the set analysis (Fig. 6). This could be a consequence of the more intensive housing conditions of dairy calves compared to beef, the use of artificial feeding equipment, or due to behaviours such as navel sucking [40, 41].

Immediately after calving, there were a large number of ASVs that were common to the microbiota of calf faeces and adult cow vagina, faeces, and teat-skin (Fig. 5), but there was little, if any, indication of these ASVs still being present in calf faeces at four-weeks of age. These results suggest that although microbes may be transferred between multiple niches on the dam and the calf faeces at calving, they do not persist, and it has been reported that the faecal microbiota of calves does not establish until after weaning [17]. Our results are in contrast to the results reported by Yeoman et al. (2018) who found operational taxonomic units (OTUs) were common to multiple sites in the calf GIT and the dam vagina, udder skin, and colostrum over the first three weeks of life, however, the degree of sharing appeared to peak within the first week and then decline for faecal samples [1]. Lima et al. (2019) also found OTUs were present in calf faeces at 3, 14 and 35 days old which were present in the faeces of the dam despite prompt separation of the calf and dam [33]. It should be noted that caution is required when comparing conclusions drawn from OTU analysis with ASV analysis, as OTU methods are less exact and more prone to errors than ASV methods [42, 43].

Overall, the most abundant family in adult faecal samples was *Ruminococcaceae*, as reported by others [9, 44], but it was present in a low relative abundance in newborn calves. After four weeks, *Ruminococcaceae* had increased in beef calves to a similar relative abundance to adult cows but only to approximately half the adult level in dairy calves. At four weeks the *Ruminococcaceae* genus with the greatest relative abundance in calves was *Faecalibacterium.* The relative abundance of *Faecalibacterium* was low (< 0.01%) at calving in both beef and dairy calves but by four weeks, it was 11.9% in beef calves but only 6.3% in dairy calves. A high prevalence of *Faecalibacterium* in the first week of life has previously been associated with higher weight gains and a reduced risk of diarrhoea in dairy calves [6].

### Vaginal microbiota

Set analysis indicated that only ASVs which were common to both the vaginal microbiota and the faecal microbiota of the adult cow were also present in the oral or faecal microbiota of calves. It is therefore difficult to unravel which of these anatomical niches is most responsible for the potential transfer of maternal microbes to the calf. Yeoman et al. (2018) observed common OTUs in both the vaginal microbiota of the dam and the gastrointestinal tract of the calf [1], although the faecal microbiota of the adult cow was not sampled. Within adult cows, the two anatomical niches which shared the greatest number of different ASVs were the vaginal and faecal microbiota. It is possible that this is the result of contamination during sampling but may also reflect genuinely similar microbial environments in cattle and other studies have also reported the microbiota of the faeces and vaginal mucosa to be compositionally similar [8, 33].

### Colostrum microbiota

There were a large number of ASVs common to the calf oral and faecal microbiota at calving that were present in dam samples including colostrum. In beef animals, these included 20 ASVs which were exclusively present in cow colostrum and the calf oral and faecal microbiota. By four-weeks, however, there were no ASVs present in calf faecal and oral microbiota that were present in the colostrum unless they were also present in other adult cow niches at calving (Fig. 5). In dairy animals, the only ASVs that were common to the faecal and oral microbiota of newborn dairy calves and dairy cow colostrum were also present in the teat skin and oral microbiota of dairy cows and by four-weeks of age very few, if any, of these ASVs were still present. There is, therefore, nothing in these results to suggest the calf gastrointestinal tract is seeded exclusively from colostrum. This is consistent with the findings reported by Klein-Jöbstl et al. (2019) [9] but in contrast to a previous study which suggested microbes in colostrum contribute to the gastrointestinal microbiota for the first weeks of life [1]. It is possible that microbes which do not persist in the gastrointestinal microbiota are still relevant to the development of the immune system; the gastrointestinal microbiota has been shown to be highly correlated with mucosal gene expression in calves, specifically genes that are related to immune function [45]. Therefore, transient changes in the gastrointestinal microbiota may have longer-lasting effects.

### Study limitations

A limitation of this study was the small number of animals included, unfortunately this is a common problem in many microbiome studies in cattle [1, 8, 9, 46–48]. The limited power reduces the scope of our study to perform robust statistical analysis which could provide more conclusive results. Additionally, the study design limited the interpretation of results specific to the influence of the dam on the microbiota of calves. The study was designed to describe the changes in cows and calves in the periparturient period on different farming systems. The beef and dairy farms used in this study employed different, but typical, approaches to calf management and therefore the opportunity existed to explore the influence of the dam, but important factors such environment, breed and diet were not able to be controlled.

Faecal swabs, and to a lesser extent oral swabs, are frequently used as the basis of exploring the microbiome of the calf gastrointestinal tract. Studies which used more invasive techniques indicate similarities between faecal samples and other gastrointestinal niches such as the colon and caecum, but little similarity to proximal intestines [1, 5, 46]. Equally, a high degree of correlation between the oral and rumen microbiota was described by Tapio et al. (2016) [47]; although oral samples in that study were collected immediately following regurgitation which was not the case in our study. Using oral and faecal samples to assess gastrointestinal microbiota is a compromise between invasive sampling, which provide the most precise results, and non-invasive sampling which allows animals to be sampled repeatedly over time in farm production settings [5]. Changes in relative abundances should be interpreted cautiously and not conflated with absolute abundances; furthermore, comparing sample composition based on family can create erroneous impressions of similarities compared to comparisons made with lower taxonomic levels. Finally, if ASVs were identified in different microbial niches in cows and calves it was inferred that this represented common, and possibly shared, microbes. However, this assertion is based on 16S rRNA sequencing which is less accurate than strain-level metagenomic techniques which have been used to more robustly demonstrate the vertical transmission of specific bacterial strains in humans [21].

## Conclusions

There were no marked differences between the development of the oral and faecal microbiota in beef or dairy calves during the first four weeks of life. This suggests that continued contact with the dam has little influence on the early maturation of oral and faecal microbiota.

The oral microbiota of calves matured more quickly than the faecal microbiota and by four-weeks of age contained similar microbes to those in the oral microbiota of adult cows. The microbiota of calf faeces changed over the first four weeks of life, but microbial constituents bore little resemblance to those in the faeces of adult cows. Any maternal influence on these changes must have occurred immediately post-partum as there were few differences between the trends observed in beef and dairy calves, despite dairy calves having limited contact with their dam.

ASVs were present in the microbiota of both the calf and dam at calving, but after four weeks more were still present in the oral microbiota of calves than their faecal microbiota. This trend was observed in both beef and dairy calves and may suggest that immediately after calving the dam shares more microbes with the calf oral microbiota than the faecal microbiota. Microbes identified in cow colostrum were also present in calf faeces at calving, but none were still present in calf faeces by four-weeks of age suggesting colostrum did not have a persistent seeding effect on the faecal microbiota of neonatal calves.

## Methods

### Farm description

Sample collection took place between February and May 2018 on the two University of Liverpool farms located in Cheshire in the United Kingdom. The dairy farm is an all year-round calving herd which milks 200 Holstein cows, three times daily, through a 12-a-side herringbone parlour. All cows and heifers are bred following artificial insemination, the majority following observed oestrus. Cows are bred with Holstein semen initially followed by beef semen if inseminations are unsuccessful. Milking cows are housed in one freestall barn on concrete cubicles with mattresses and sawdust. Cows in the first two weeks of lactation are loose housed on a straw yard and milked twice daily. Dry cows are kept in far-off and close-up groups, both on straw yards, with cows moving into the close-up group approximately 30 days prior to expected calving; pregnant heifers join the close-up dry cow group at the equivalent timepoint. All cows are dried off 60 days prior to expected calving with internal test sealant (Orbeseal Dry Cow Intramammary Suspension, Zoetis) and, depending on their clinical mastitis and somatic cell count history, antibiotic dry cow therapy (Ubro Red Dry Cow Intramammary Suspension, Boehringer Ingelheim).

Cows are moved into an adjacent group calving pen during the first stage of parturition which is re-bedded with straw between animals. Calves are fed 3 L fresh colostrum from the dam via an oesophageal feeding tube within three hours of birth and the calf’s navel is dipped into iodine solution. Calves are removed from the dam within six hours of birth and housed in individual calf hutches for ten days followed by housing in groups of six calves until weaning at ten weeks old. Calves are fed artificial milk replacer twice daily corresponding to 10% of bodyweight, with hay, starter feed and fresh water freely available.

The beef farm is a herd of 16 Hereford cows with a nine-week calving period which begins on 1st April. Cows are initially bred with fixed-timed artificial insemination and then run with a Hereford bull. Cows are loose housed on straw yards over winter and fed grass silage; they are grazed over summer. Cows calve outside across multiple paddocks with three or fewer cow-calf pairs per paddock; cow-calf pairs are mixed once all calves are over 4 weeks old. Neonatal calves are monitored to ensure they have been seen suckling the dam and the calf’s navel is dipped into iodine solution within six hours of birth.

### Enrolment

All primiparous and multiparous cows were enrolled if they had an expected calving date in April 2018, unless the cow was lame in which case they were excluded. Cow-calf pairs were subsequently excluded from the study if parturition had required intervention from farm staff, more than a single calf had been born, cow compliance affected sample collection at any stage, the time between calving the first sampling timepoint exceeded 12 h, or if the cow or calf had required antibiotic treatment during the study period, with the exception of antibiotic dry cow therapy. Additionally, only dairy cows which had a Holstein calf were included. The number of samples which could be processed was limited to five cow-calf pairs from each farm, so sample collection ceased once this number had been reached.

### Sample collection

Five anatomical niches were sampled in each adult cow: teat-skin, milk, vaginal mucosa, faeces, and oral mucosa; in calves only oral mucosa and faeces were sampled. Adult dairy cows were sampled at drying off, approximately eight weeks prior to calving, and adult beef cows were sampled four weeks prior to calving. Adult cows were sampled again within 12 h of calving along with faecal and oral swabs from their calves; samples were collected from cows and calves for a final time 3–4 weeks later.

Teat-skin around the teat orifice was sampled in all cows: dry paper towel was used to remove gross contamination and a single swab was rubbed firmly around teat orifice of all four teats sequentially. The vagina was sampled in all cows: dry paper towel was used to remove gross contamination from the vulva which was then parted to allow the swab to be inserted to the midpoint of the vagina, rubbed against the vaginal mucosa for several seconds and removed without contact with the vulva. Milk or colostrum samples were collected from all cows at each time point with the exceptions of beef cows prior to calving because they were not lactating. Prior to milk or colostrum collection, each teat was cleaned with dry paper towel to remove gross contamination, 3–4 streams of milk was expressed and discarded, and pre-milking teat disinfectant was applied to each teat. After 30 s each teat was dried with paper towel and the teat end scrubbed with gauze soaked in surgical spirit. Gloves were changed at this stage and an additional three streams of milk discarded; milk was then expressed into a sterile Falcon tube. Samples from each quarter were collected and stored separately. The oral cavity was sampled in cows and calves: the mouth was opened, and the swab rubbed against the dorsal tongue for several seconds until it was soaked with saliva. Finally, faecal swabs were collected from all cows and calves by inserting the swab into the rectum until it was soaked in faeces. The order of sample collection was always maintained, starting with the cow: teat-skin, milk or colostrum, vaginal mucosa, faeces, and finally the oral mucosa. Samples were then collected from the calf, first the oral swab followed by the faecal. All samples were taken by the same veterinary surgeon. Clean gloves were worn for each sample collected; the end of each sterile cotton swab was snapped into a pre-labelled, sterile Eppendorf tube. All samples were placed into ice and then transferred to − 80 °C storage within two hours.

### DNA extraction

Following storage at − 80 °C for a maximum of 2 months, samples were thawed at room temperature immediately prior to extraction. DNA extraction was conducted in batches by sample type and a negative control was included in each DNA extraction batch, therefore a total of eight DNA extraction controls were included. All extractions were completed using the Thermo Fisher PureLink™ Microbiome DNA Purification Kit (ThermoFisher Scientific) which utilizes chemical, heat and bead-beating cell lysis prior to purification. The swab was used directly as the source for DNA extractions from samples of the teat-skin, vagina mucosa, oral mucosa and faeces (i.e. the end of the swab was placed directly into the first reagent). The milk and colostrum samples were first pooled to create a composite sample, with equal volumes from all four quarters, and a 500 μL aliquot of this was used for the DNA extraction. The quantity of DNA in each sample was measured following extraction using a Qubit 2.0 fluorometer (Invitrogen).

### Amplicon production and bioinformatic analysis

Full details of the following steps are provided the Supplementary methods but are summarised below. Previously described primers [49] were used to amplify the hypervariable V4 region of bacterial 16S rRNA. A total of 35 cycles were used for each sample which included incorporation of barcodes as described in the Illumina Nextera protocol. The amplicon libraries were sequenced on an Illumina MiSeq platform to generate 2 × 250 bp paired end reads. The raw sequence pairs for each sample were processed for analysis using a custom pipeline based on QIIME2 2018.11. Samples were assessed for quality control which involved exclusion of short and chimeric sequences, and denoising. Amplicon sequence variants (ASVs) were identified, the phylogenetic relationship among the identified ASVs was defined and ASVs were taxonomically assigned. Descriptive analyses of sequencing results and taxonomic classification are presented using means/standard deviations and median/interquartile ranges calculated in Microsoft Excel.

Samples were normalised at a rarefaction threshold of 12,000 sequences. Alpha-diversity was assessed primarily by calculating the Shannon richness index [50]. Beta-diversity was initially explored by calculating the Bray-Curtis dissimilarity, and Weighted and Unweighted UniFrac distances which were calculated in QIIME [51, 52]. The principal coordinates analysis (PCoA) were plotted using EMPeror [53]. The compositional differences between non-rarefied samples were assessed by permutational analysis of variance (PERMANOVA) using R (R Core Team, Vienna, Austria).

Gneiss analysis was used to visualise ASV abundance between sample groups [54]. ASVs present in an unfiltered ASV table of all samples was used to define ASVs which were “present” in each sample group if its relative abundance across all samples of that group was greater than 0.01%. Intersections between sets of ASVs were visualised using UpSet plots [55] and plotted using Intervene [56]. The taxonomy of the ASVs present in these intersections were examined and considered in the context of their relative abundance in each sample type.

## Supplementary information

**Additional file 1: Supplementary Figure 1.** The relative abundances of the four most prevalent phyla in oral (a) and faecal (b) samples. Beef and dairy animals are plotted side by side, samples from calves are compared between “Calving” (within 12 h of parturition) and “4 weeks” (four-weeks of age). Samples from adult cows are displayed as an average across all three timepoints (six to eight weeks prepartum, within 12 h of parturition, and four-weeks postpartum). The number of individual animals in each group: calf samples *N* = 5 and adult cow samples *N* = 15.

**Additional file 2: Supplementary Figure 2.** Principal Coordinate Analysis (PCoA) of unweighted UniFrac distances at each timepoint. Beef animals are represented by circles and dairy animals by triangles. All three timepoints (six to eight weeks prepartum, within 12 h of parturition, and four-weeks postpartum) are displayed. The two plots display the same data but with different y-axes to display the three-dimensional relationship between datapoints.

**Additional file 3: Supplementary Figure 3.** UpSet plots of common assigned sequence variants (ASVs) between oral and faecal samples. Beef (a) and dairy (b) animals are displayed separately. Oral and faecal samples from cows and calves at “Pre-calving” (4–8 weeks prepartum), “Calving” (within 12 h of parturition) and 4wk (4 weeks postpartum) are displayed. Only ASVs with an overall abundance across all samples of greater than 0.01% are included, the 30 intersections which involve the greatest number of ASVs are displayed. The number of individual animals in each group: *N* = 5. Taxonomy of each intersection is detailed in Supplementary Table 6 and 7.

**Additional file 4: Supplementary Figure 4.** Dendrogram heatmap of log abundance of assigned sequence variants (ASVs) in each sample. Samples are labelled by timepoint “PrC” (4–8 weeks prior to parturition), “PoC” (within 12 h of parturition) and “Wk4” (4 weeks after parturition); then as “Cow” or “Calf” depending on the age of the animal; then by anatomical site sampled: “Fae” (faeces), “Milk”, “Vag” (vaginal mucosa), “Or” (oral mucosa), “Teat” (teat skin) or “Colos” (colostrum); and finally by animal type: “B” (beef) or “D” (dairy). The number of individual animals in each group: N = 5.

**Additional file 5: Supplementary Table 1.** Summary of sequencing and alpha-diversity analysis for each sample. ASV = assigned sequence variants. Only samples which exceeded the rarefaction threshold of 12,000 reads were included in the Shannon diversity index calculation.

**Additional file 6: Supplementary Table 2.** The taxonomic assignment of the ASVs in the intersections of beef animals which are displayed in Fig. 5.

**Additional file 7: Supplementary Table 3.** The taxonomic assignment of the ASVs in the intersections of dairy animals which are displayed in Fig. 5.

**Additional file 8: Supplementary Table 4.** The taxonomic assignment of the ASVs in the intersections of beef animals which are displayed in Fig. 6.

**Additional file 9: Supplementary Table 5.** The taxonomic assignment of the ASVs in the intersections of dairy animals which are displayed in Fig. 6.

**Additional file 10: Supplementary Table 6.** The taxonomic assignment of the ASVs in the intersections of beef animals which are displayed in Supplementary Fig. 3.

**Additional file 11: Supplementary Table 7.** The taxonomic assignment of the ASVs in the intersections of dairy animals which are displayed in Supplementary Fig. 3.

**Additional file 12: Additional Data 1.** Summary statistics of the relative abundance of family in each sample.

**Additional file 13.** Detailed methods.

## Data Availability

The datasets generated and analysed during the current study are available at https://www.ncbi.nlm.nih.gov/bioproject/625876 (Accession Number: PRJNA625876).

## Abbreviations

ASV: Amplicon sequence variant
DNA: Deoxyribonucleic acid
IQR: Inter-quartile range
OTU: Operational taxonomic unit
PCoA: Principal coordinates analysis
PCR: Polymerase chain reaction
PERMANOVA: Permutational analysis of variance
rRNA: Ribosomal ribonucleic acid
SD: Standard deviation
