# Supplementary figures and images for: Maternal influences on oral and faecal microbiota maturation in neonatal calves in beef and dairy production systems
Source: Anim Microbiome. 2020 Sep 7;2:31. doi: 10.1186/s42523-020-00049-1 (PMC7807724; doi:10.1186/s42523-020-00049-1)

a) Oral

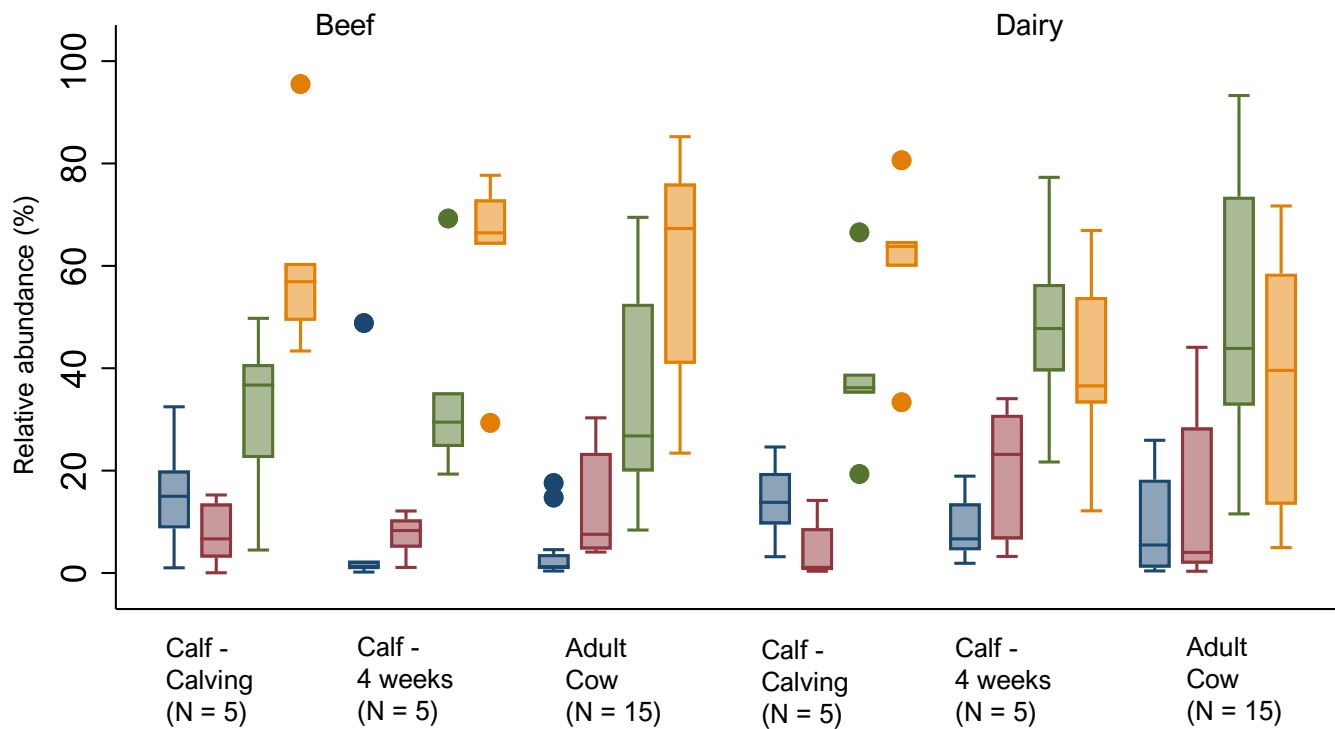

b) Faecal

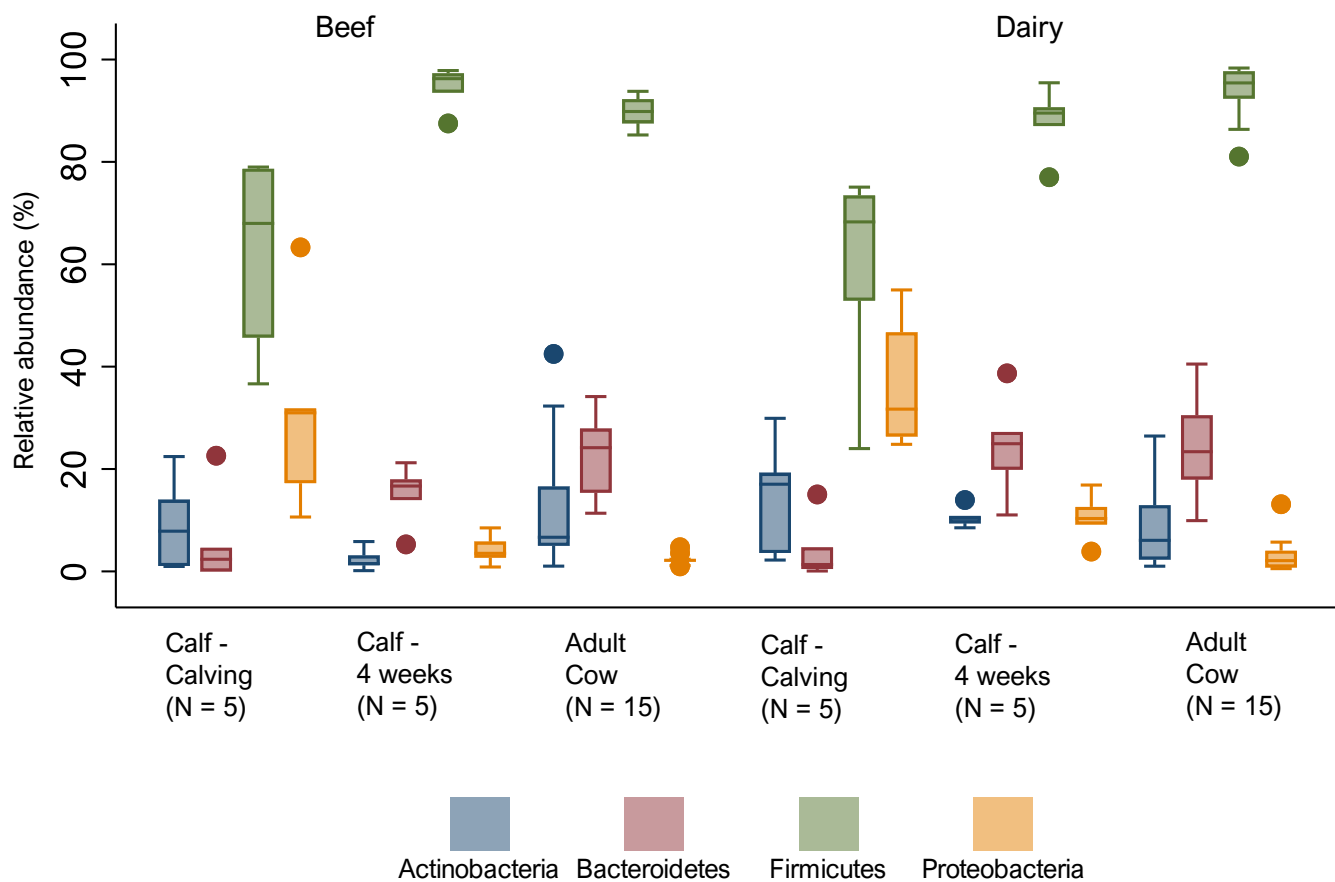

Supplement: Supplementary file 1 — Additional file 1: Supplementary Figure 1. The relative abundances of the four most prevalent phyla in oral (a) and faecal (b) samples. Beef and dairy animals are plotted side by side, samples from calves are compared between “Calving” (within 12 h of parturition) and “4 weeks” (four-weeks of age). Samples from adult cows are displayed as an average across all three timepoints (six to eight weeks prepartum, within 12 h of parturition, and four-weeks postpartum). The number of individual animals in each group: calf samples N = 5 and adult cow samples N = 15. [file 42523_2020_49_MOESM1_ESM.pdf]

PC2 (9.9%)

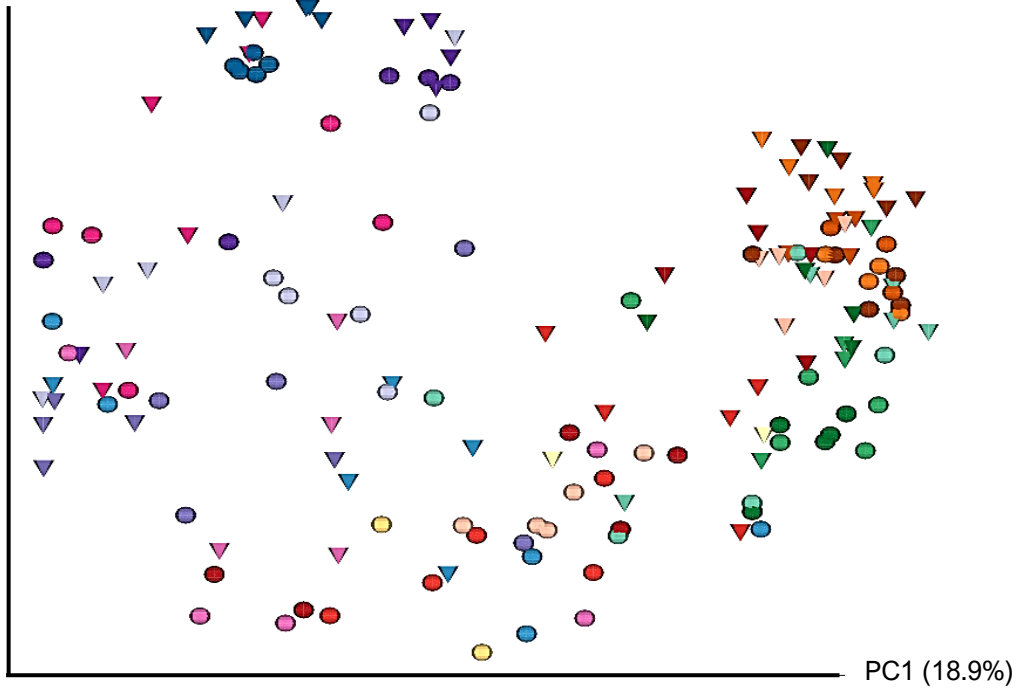

PC3 (7.2%)

▼ Triangles: dairy cows    ● Circles: beef cows

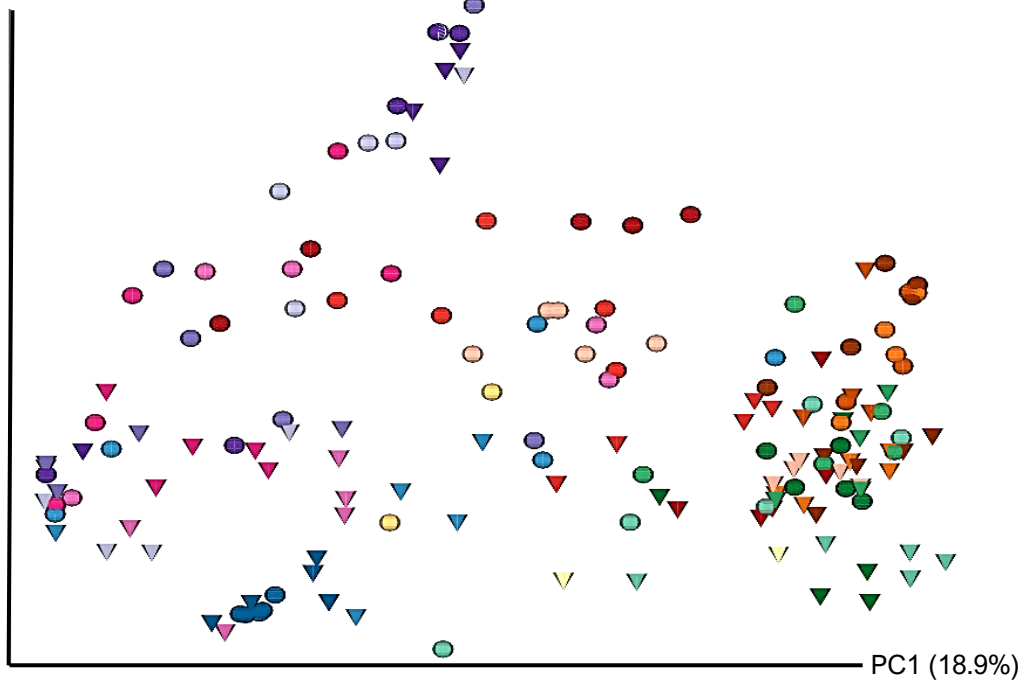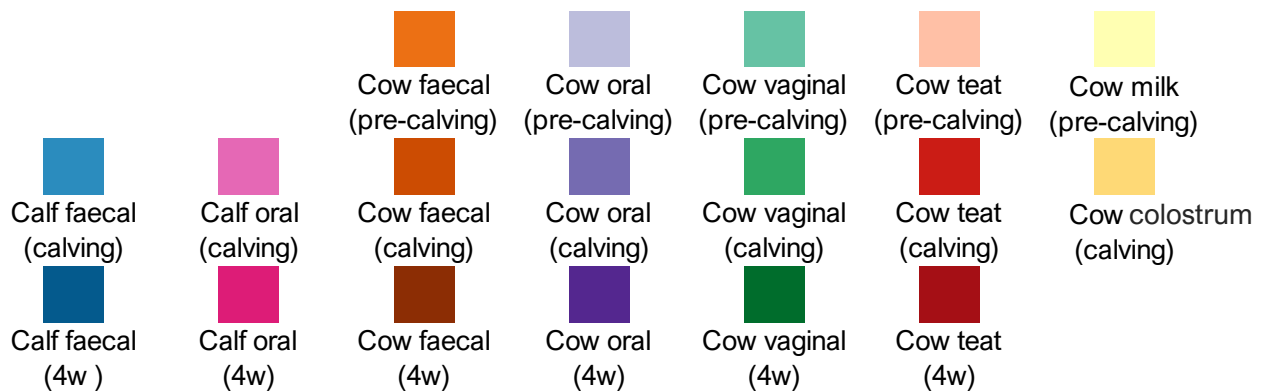

Supplement: Supplementary file 2 — Additional file 2: Supplementary Figure 2. Principal Coordinate Analysis (PCoA) of unweighted UniFrac distances at each timepoint. Beef animals are represented by circles and dairy animals by triangles. All three timepoints (six to eight weeks prepartum, within 12 h of parturition, and four-weeks postpartum) are displayed. The two plots display the same data but with different y-axes to display the three-dimensional relationship between datapoints. [file 42523_2020_49_MOESM2_ESM.pdf]

a) Beef

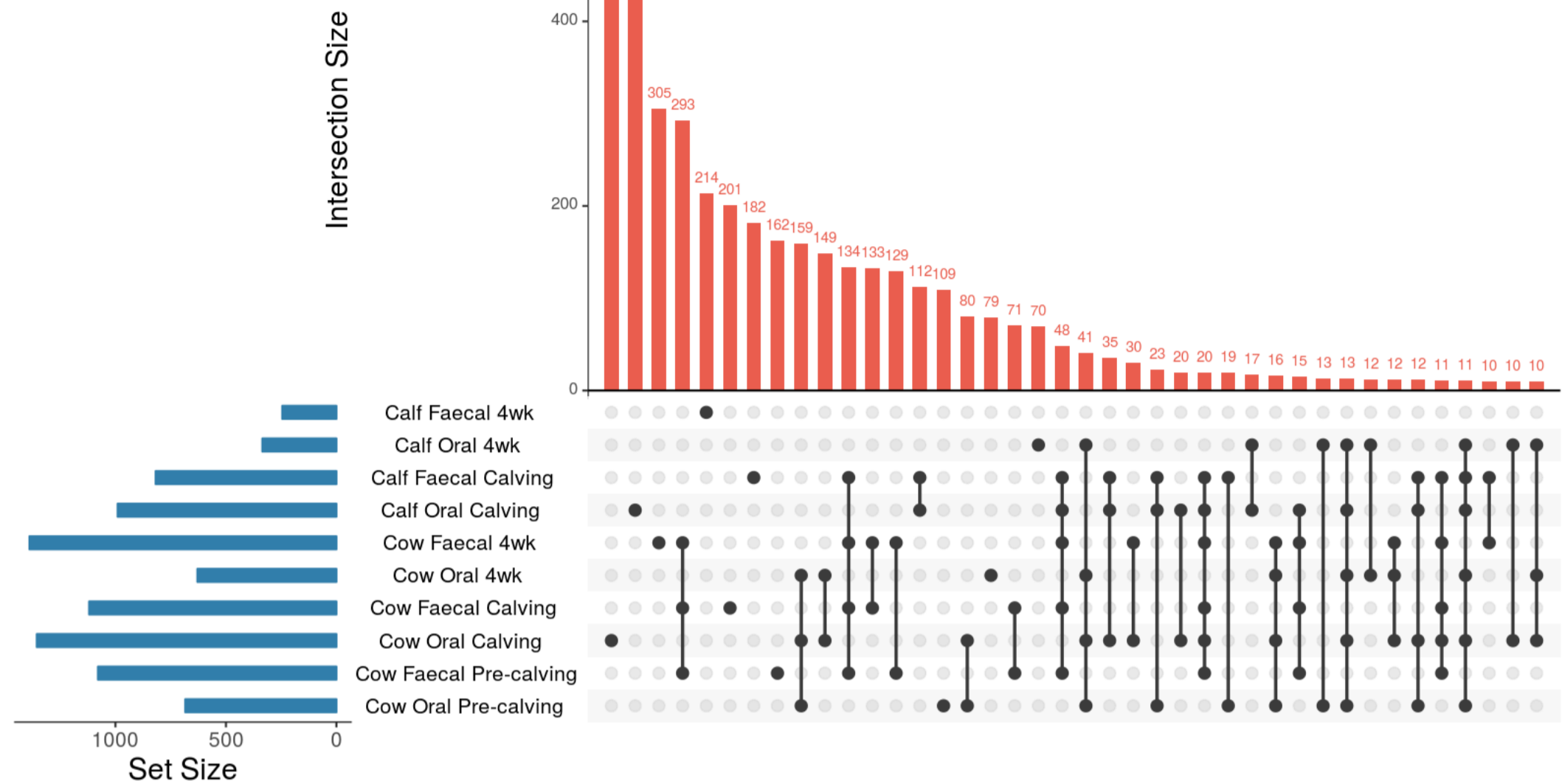

## b) Dairy

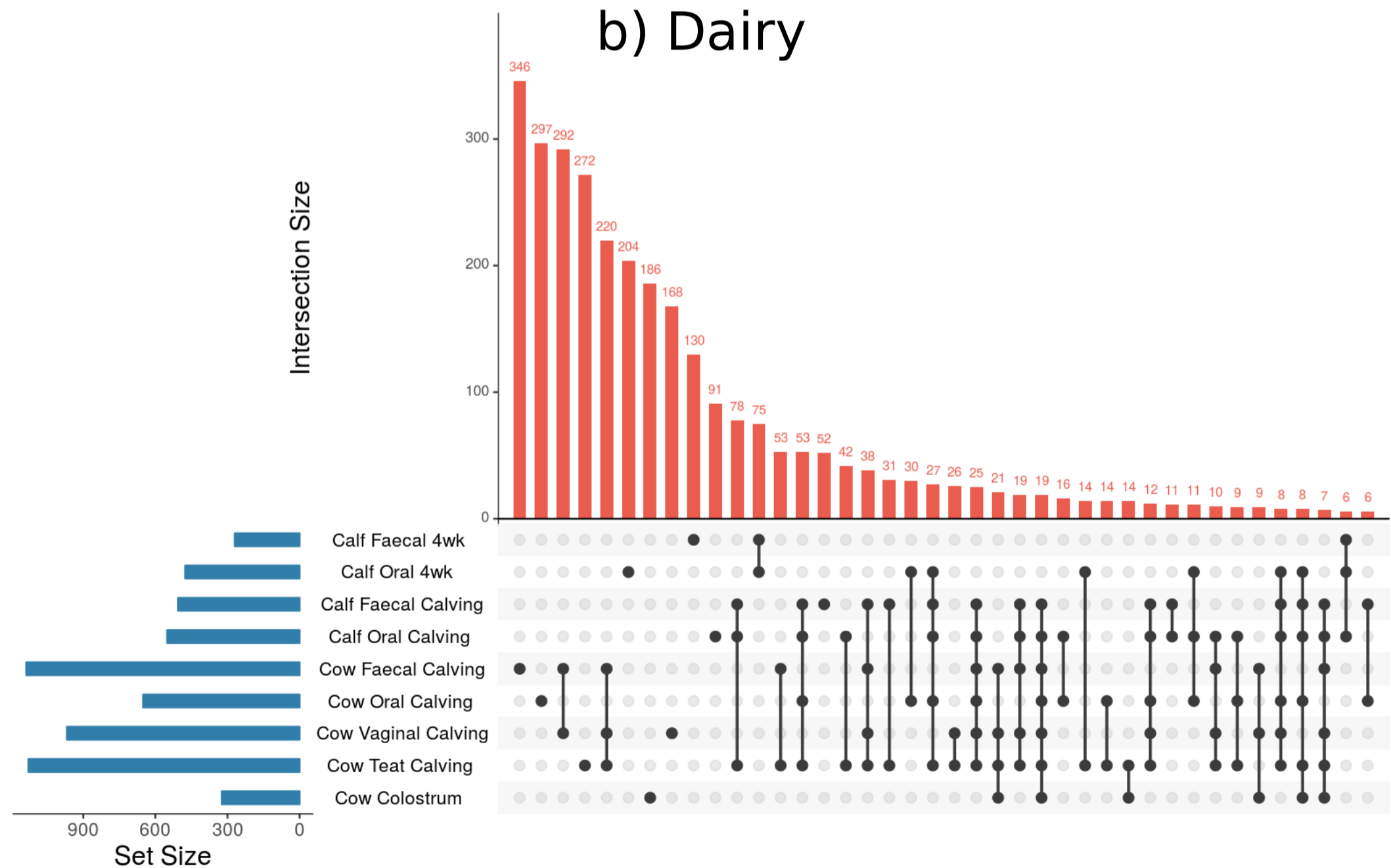

Supplement: Supplementary file 3 — Additional file 3: Supplementary Figure 3. UpSet plots of common assigned sequence variants (ASVs) between oral and faecal samples. Beef (a) and dairy (b) animals are displayed separately. Oral and faecal samples from cows and calves at “Pre-calving” (4–8 weeks prepartum), “Calving” (within 12 h of parturition) and 4wk (4 weeks postpartum) are displayed. Only ASVs with an overall abundance across all samples of greater than 0.01% are included, the 30 intersections which involve the greatest number of ASVs are displayed. The number of individual animals in each group: N = 5. Taxonomy of each intersection is detailed in Supplementary Table 6 and 7. [file 42523_2020_49_MOESM3_ESM.pdf]

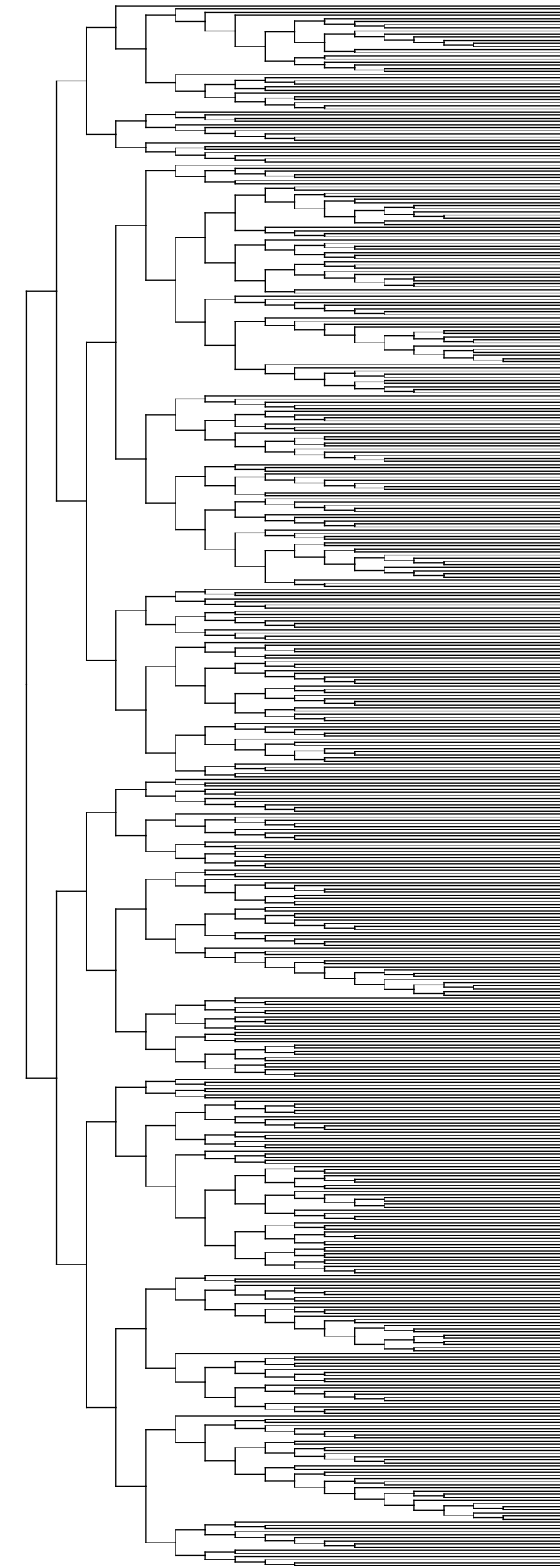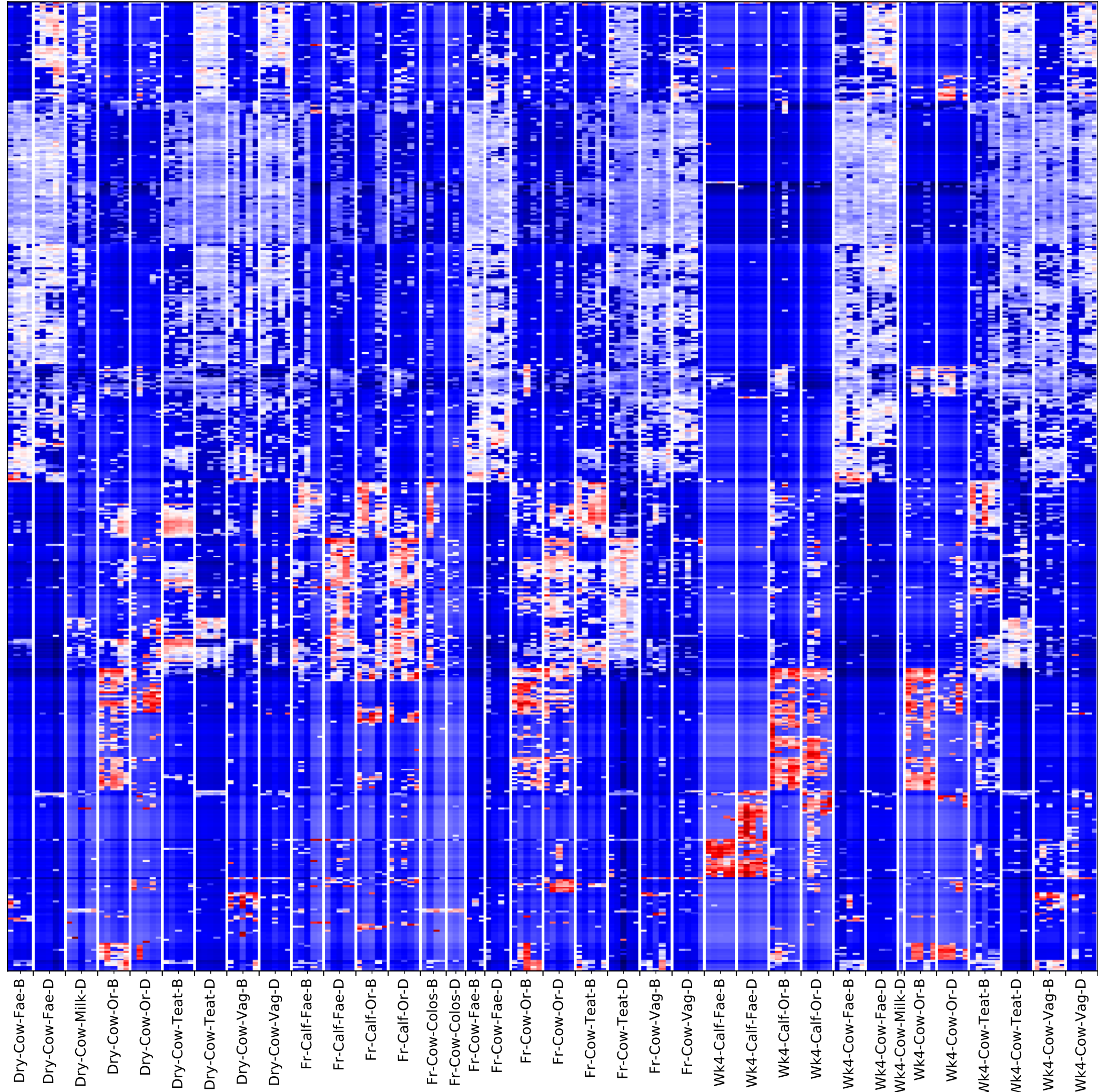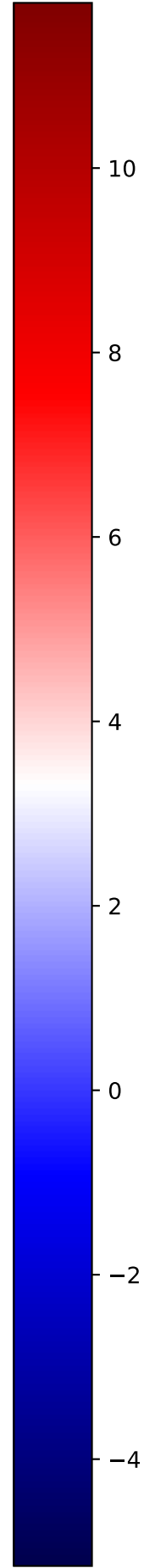

Supplement: Supplementary file 4 — Additional file 4: Supplementary Figure 4. Dendrogram heatmap of log abundance of assigned sequence variants (ASVs) in each sample. Samples are labelled by timepoint “PrC” (4–8 weeks prior to parturition), “PoC” (within 12 h of parturition) and “Wk4” (4 weeks after parturition); then as “Cow” or “Calf” depending on the age of the animal; then by anatomical site sampled: “Fae” (faeces), “Milk”, “Vag” (vaginal mucosa), “Or” (oral mucosa), “Teat” (teat skin) or “Colos” (colostrum); and finally by animal type: “B” (beef) or “D” (dairy). The number of individual animals in each group: N = 5. [file 42523_2020_49_MOESM4_ESM.pdf]
