# Supplementary material for: Maternal influences on oral and faecal microbiota maturation in neonatal calves in beef and dairy production systems
Source: Anim Microbiome. 2020 Sep 7;2:31. doi: 10.1186/s42523-020-00049-1 (PMC7807724; doi:10.1186/s42523-020-00049-1)
Supplement: Supplementary file 13 — Additional file 13. Detailed methods. [file 42523_2020_49_MOESM13_ESM.docx]

**Detailed methods**

*Amplicon production*

Previously described primers [1] were used to amplify the hypervariable V4 region of bacterial 16S rRNA. Primers:

F: 5'ACACTCTTTCCCTACACGACGCTCTTCCGATCTNNNNNGTGCCAGCMGCCGCGGTAA3'

R: 5'GTGACTGGAGTTCAGACGTGTGCTCTTCCGATCTGGACTACHVGGGTWTCTAAT3'

NEBNext^®^ High-Fidelity 2X PCR Master Mix (New England Biolabs, France) was used in both PCRs. 5µl of DNA entered a first round PCR with conditions: denaturation for 10 seconds at 98°C, annealing for 30 seconds at 50°C, extension for 30 seconds at 70°C for 20 cycles and finally a 10 minute extension at 72°C. Samples were then purified with AMPure SPRI Beads before entering the second PCR with barcodes incorporated for sample identification. The barcode sequences are the same as those described in the Illumina Nextera protocol. The general sequences of the forward and reverse primers are illustrated below. The 8 bp barcode is underlined.

N501 f:

5'AATGATACGGCGACCACCGAGATCTACACTAGATCGCACACTCTTTCCCTACACGACGCTC3'

N701 r:

5'CAAGCAGAAGACGGCATACGAGATTCGCCTTAGTGACTGGAGTTCAGACGTGTGCTC3'

15 cycles of second round PCR were performed using the same conditions as above for a total of 35 cycles. Samples were purified again with AMPure SPRI Beads before being quantified using Qubit and assessed using the Fragment Analyzer. Successfully generated amplicon libraries were taken forward.

Final libraries were pooled in equimolar amounts using the Qubit and Fragment Analyzer data and size selected on the Pippin prep using a size range of 300-600bps. The quantity and quality of each pool was assessed by Bioanalyzer and subsequently by qPCR using the Illumina Library Quantification Kit from Kapa on a Roche Light Cycler LC480II according to the manufacturer's instructions. The 16S rRNA gene libraries were sequenced on an Illumina MiSeq platform with version 2 chemistry using sequencing by synthesis technology to generate 2 x 250 bp paired end reads. To help balance the complexity of the amplicon library 15% PhiX was spiked in.

*Bioinformatic analysis*

Base-calling and de-multiplexing of indexed reads was performed by CASAVA version 1.8.2 (Illumina) to produce 195 samples sequence files, in FASTQ format. The raw FASTQ files were trimmed to remove Illumina adapter sequences using Cutadapt version 1.2.1 [2]. The reads were further trimmed to remove low quality bases. After trimming, reads shorter than 20 bp were removed. The raw sequence pairs for each sample, were processed for analysis using a custom pipeline based on QIIME2 2018.11 [3]. The SILVA (version 123) was used throughout the analysis. The raw reads were subjected to a “Cutadapt” trimming step to remove PCR primer sequences that could potentially introduce an artificial level of complexity in the samples.

The amplicon sequence variants (ASVs) are defined as the biological sequences present in samples prior to the PCR amplification step. The “dada2” process was performed using the denoise-paired QIIME2 plug-in; it includes the following steps:

1. Excluding sequences shorter than the selected thresholds, prior to denoising.

2. De-noising step.

3. Merge step to pair the denoised forward and reverse reads.

4. Remove the chimeric sequences derived by the PCR step.

5. Remove the sequences derived by PhiX phage (added to sequence library to increase sequence complexity).

After the de-noising step, these sequences were further investigated to define the phylogenetic relationship among the identified ASVs. To do this, the alignment of the sequences was obtained using MAFFT [4]. The phylogenetic tree was built using the resulting alignment. The uninformative regions of the obtained alignment were masked before the inference of the tree using the FastTree tool [5]. As a final step, the tree was converted from an unrooted to a rooted tree by predicting the branch mid points. The tree construction was performed using “align mafft”, “alignment mask”, “phylogeny fasttree”, and “midpoint-root” QIIME2 plug-ins. The taxonomy assignment of the identified ASVs, was performed by using the sk-learn tool [6].

The negative control samples were excluded from alpha-diversity, beta-diversity, and differential abundance analysis. The sequencing depth of all samples were explored using the ‘Shannon’ [7] richness index plotted as a rarefaction curve. The diversity analyses (both alpha- and beta- diversity) were performed using the “diversity core-metrics phylogenetic” QIIME2 plug-in, following normalisation of the abundance counts by rarefaction at a threshold of 12,000 sequences. Samples with a lower number of sequences than this threshold were excluded from the analysis.

The richness diversity between samples was investigated using the Shannon richness index which was calculated for each sample type and then further split by sample timepoint, animal age, animal type and sample type. To study how the taxonomic composition changed between samples, the rarefied abundance was used to build pairwise sample distance matrices, using the Bray-Curtis [8] and the Weighted and Unweighted UniFrac dissimilarity measures [9]. These diversity matrices were produced by the “diversity core-metrics-phylogenetic” QIIME2 plug-in. The Principal Coordinate Analysis (PCoA) methods were then used and plotted using EMPeror [10]. The compositional differences between non-rarefied samples were assessed by permutational analysis of variance (PERMANOVA) using R (R Core Team, Vienna, Austria).

Gneiss analysis was used to visualise ASV abundance between sample groups [11]. To facilitate interpretation of results, the most abundant 1000 ASVs were included in the analysis by filtering ASVs with a total frequency of less than 1045. This removed 13,125 ASVs from the analysis. Gneiss was run using the gneiss plugin (https://biocore.github.io/gneiss/). First, a dendrogram of ASVs was created using Ward's hierarchical clustering. This method clusters ASVs which co-occur in samples together. An isometric log abundance of each ASV was calculated using the ilr-transform command. Results were visualised through a dendrogram heatmap (Supplementary Figure 4).

ASVs present in an unfiltered ASV table of all samples was used to define ASVs which were “present” in each sample group. An ASV was defined as “present” in a sample group if its relative abundance across all samples was higher than 0.01%. Once lists of ASVs present in each sample group were compiled, intersections between sets of ASVs were visualised using UpSet [12].

1. Caporaso JG, Lauber CL, Walters WA, Berg-Lyons D, Lozupone CA, Turnbaugh PJ, et al. Global patterns of 16S rRNA diversity at a depth of millions of sequences per sample. Proc Natl Acad Sci. 2011;108 Supplement_1:4516–22. doi:10.1073/pnas.1000080107.

2. Martin M. Cutadapt removes adapter sequences from high-throughput sequencing reads. EMBnet.journal. 2011;17:10.

3. Bolyen E, Rideout JR, Dillon MR, Bokulich NA, Abnet CC, Al-Ghalith GA, et al. Reproducible, interactive, scalable and extensible microbiome data science using QIIME 2. Nature Biotechnology. 2019;37:852–7.

4. Katoh K, Standley DM. MAFFT multiple sequence alignment software version 7: Improvements in performance and usability. Mol Biol Evol. 2013;30:772–80.

5. Price MN, Dehal PS, Arkin AP. FastTree 2 - Approximately maximum-likelihood trees for large alignments. PLoS One. 2010;5:e9490.

6. Pedregosa F, Varoquaux G, Gramfort A, Michel V, Thirion B, Grisel O, et al. Scikit-learn: Machine learning in Python. J Mach Learn Res. 2011;12:2825–30.

7. Shannon CE. A Mathematical Theory of Communication. Bell Syst Tech J. 1948;27:379–423. doi:10.1002/j.1538-7305.1948.tb01338.x.

8. Bray JR, Curtis JT. An Ordination of the Upland Forest Communities of Southern Wisconsin. Ecol Monogr. 1957;27:325–49. doi:10.2307/1942268.

9. Lozupone C, Knight R. UniFrac: A new phylogenetic method for comparing microbial communities. Appl Environ Microbiol. 2005;71:8228–35.

10. Vázquez-Baeza Y, Pirrung M, Gonzalez A, Knight R. EMPeror: a tool for visualizing high-throughput microbial community data. Gigascience. 2013;2:16. doi:10.1186/2047-217X-2-16.

11. Morton JT, Sanders J, Quinn RA, McDonald D, Gonzalez A, Vázquez-Baeza Y, et al. Balance Trees Reveal Microbial Niche Differentiation. mSystems. 2017;2. doi:10.1128/mSystems.00162-16.

12. Lex A, Gehlenborg N, Strobelt H, Vuillemot R, Pfister H. UpSet: Visualization of intersecting sets. IEEE Trans Vis Comput Graph. 2014;20:1983–92.
